# Chronic detachable headphones for acoustic stimulation in freely moving animals

**DOI:** 10.1016/j.jneumeth.2010.03.017

**Published:** 2010-05-30

**Authors:** Fernando R. Nodal, Peter Keating, Andrew J. King

**Affiliations:** Department of Physiology, Anatomy and Genetics, University of Oxford, Oxford OX1 3PT, United Kingdom

**Keywords:** Spatial hearing, Sound localization, Sound lateralization, Headphone, Ferret, Binaural, Interaural level difference, Bone cement

## Abstract

A growing number of studies of auditory processing are being carried out in awake, behaving animals, creating a need for precisely controlled sound delivery without restricting head movements. We have designed a system for closed-field stimulus presentation in freely moving ferrets, which comprises lightweight, adjustable headphones that can be consistently positioned over the ears via a small, skull-mounted implant. The invasiveness of the implant was minimized by simplifying its construction and using dental adhesive only for attaching it to the skull, thereby reducing the surgery required and avoiding the use of screws or other anchoring devices. Attaching the headphones to a chronic implant also reduced the amount of contact they had with the head and ears, increasing the willingness of the animals to wear them. We validated sound stimulation via the headphones in ferrets trained previously in a free-field task to localize stimuli presented from one of two loudspeakers. Noise bursts were delivered binaurally over the headphones and interaural level differences (ILDs) were introduced to allow the sound to be lateralized. Animals rapidly transferred from the free-field task to indicate the perceived location of the stimulus presented over headphones. They showed near perfect lateralization with a 5 dB ILD, matching the scores achieved in the free-field task. As expected, the ferrets’ performance declined when the ILD was reduced in value. This closed-field system can easily be adapted for use in other species, and provides a reliable means of presenting closed-field stimuli whilst monitoring behavioral responses in freely moving animals.

## 1 Introduction

Studies of auditory processing require precise control over stimulus presentation. This is easily attainable in anesthetized and un-anesthetized head-restrained preparations (Edeline et al., 1999; Fritz et al., 2007), which facilitate the measurement of responses to multiple presentations of the same stimulus. By contrast, in freely moving animals, the position relative to the head of fixed sound sources may vary from trial to trial, even when animals have been trained to stand in a certain location during stimulus presentation. Small discrepancies in relative speaker location might not be an issue in some tasks, such as the detection or discrimination of location-independent parameters like frequency or timbre. However, for studies of sound localization, variations in head position will alter the spectral shape cues and the magnitude of the interaural differences that provide the basis for determining sound-source direction (Blauert, 1997; King et al., 2001), thereby confounding measurements of spatial sensitivity.

Although auditory detection and discrimination tasks can be carried out successfully in head-restrained animals (Fritz et al., 2007), this is not the case for measurements of sound localization, where the accuracy of sound-evoked gaze shifts is impaired if the head is unable to move (Tollin et al., 2005). There is therefore a need to be able to present stimuli over headphones, both to ensure stable and reliable stimulus presentation without restricting behavioral responses and to facilitate independent control of spatial cues that normally co-vary, thereby allowing assessment of the contribution made by each cue to spatial perception (Wightman and Kistler, 1992; Jin et al., 2004) and to the response properties of auditory neurons (Tollin and Yin, 2002; Campbell et al., 2006, 2008).

Although closed-field presentation is very commonly used when recording from auditory neurons in anesthetized animals, this is much more difficult in the awake condition due to variability in the placement of earphones both during and between recording sessions. Moreover, the potential stress induced by fitting them and the resulting tactile stimulation of the face and ears may further compromise the experiment. Animals can be trained to accept such manipulations, but this is time consuming and normally requires a larger cohort of animals to undergo initial training in order to screen their suitability for wearing such devices (Wakeford and Robinson, 1974).

A potential way of reducing these problems is to position earphones via a permanent implant mounted on the animal's skull, but this approach has so far been restricted to animals, such as cats (Beitel, 1997) and barn owls (Hausmann et al., 2009), with relatively large heads, and which were also unable to carry out their normal locomotor behavior. Chronic neural recording devices have been used successfully in a range of species (e.g. Edeline et al., 1999; Fee and Leonardo, 2001; Lu et al., 2001; Dobbins et al., 2007), and are typically constructed by embedding custom-made devices in dental or bone cement. However, because these embedding cements have poor adhesive properties, it is necessary to use some form of anchoring system to provide a stronger bond between the skull and the implant. Traditionally, self-tapping screws are used for this, although these are not always suitable due to the thickness of the skull and can also pose a risk of brain damage if they protrude inside the cranium. Consequently, it is important to consider less invasive materials as a means of securing cranial implants (Okumura et al., 2007).

To address these issues in the ferret, a species that is widely used in behavioral studies of auditory processing (e.g. Kavanagh and Kelly, 1987; Fritz et al., 2007; Nodal et al., 2008), we have designed a lightweight headphone system that can be easily attached to a chronic implant when required. We have also refined the construction and fixation of the chronic implant onto the skull, thereby greatly reducing the cost to each animal.

## 2 Materials and methods

All procedures involving animals were performed following local ethical review committee approval and under licence from the UK Home Office in accordance with the Animal Scientific Procedures Act (1986). Seven female adult pigmented ferrets (*Mustela putorius furo*) from our breeding colony were used in this study.

### 2.1 Animal welfare

Ferrets were housed in standard laboratory cages in small groups of two or three animals. The cages were equipped with different objects such as balls, plastic tubes and shelters for the animals to play with. At least twice a week, animals were allowed to explore outside their cages and interact with other ferrets. During the behavioral testing periods, which each lasted a maximum of 14 consecutive days, the animals received free access to their usual dry food in their cages but were only provided with drinking water during the two or three daily training sessions in the test apparatus. If the total daily volume of water consumed during these sessions was <60 ml/kg, which we have shown to be the average daily water consumption by ferrets in our colony, supplementary fluid was provided at the end of each day's testing in the form of a puree comprising ground food pellets and an appropriate amount of water. Body weights were recorded daily and compared to the baseline weight for each animal determined before the start of the water regulation paradigm. The maximum weight drop allowed was 2SD below this mean baseline weight. Individual mean weights and their SD were calculated from weekly measurements taken since the animals were 3 months old. Between each 14-day testing period, animals were allowed breaks of ≥4 days, during which they were given *ad libitum* access to water.

### 2.2 Behavioral setup

The auditory discrimination task was carried out in a metallic mesh (13 mm opening) cage with a solid plastic floor (55 cm × 43 cm × 41 cm) enclosed with acoustic foam (MelaTech; Hodgson & Hodgson Ltd., Melton Mowbray, UK). Each animal was trained to make behavioral responses by inserting its nose into a poke-hole containing a spout that delivered a specified amount of water (typically 150–300 μl per trial). There were three such spout locations, a ‘center spout’ in the middle of one of the short sides of the chamber and two ‘response spouts’, one of which was located on each the lateral sides. Infrared LEDs were used to produce beams of light spanning the entrance to each poke-hole, the status of which was monitored continuously by photodiodes connected to a real time processor (RP2; Tucker-Davis Technologies, Alachua, FL). Poking behavior readily interrupted these beams, enabling responses to be registered. All electronic circuits were constructed using standard components (RS Components, Corby, UK) and a design similar to that used by Walker et al. (2009). A loudspeaker (FRS 8; Visaton, Crewe) was located over each of the two response spouts for free-field stimulus presentation.

All stimuli were broadband noise bursts of 200 ms duration, generated afresh on each trial at sound levels varying (in 5 dB steps) from 70 to 80 dB SPL using Tucker-Davis Technologies System 3 hardware. We used 200 ms as this matches the latency of head-orienting movements in the ferret (Nodal et al., 2008), avoiding the need to adjust the spatial cue values provided over the headphones once the head starts to move. The use of longer stimuli would, however, require that such feedback is provided. Stimulus presentation and behavioral procedures were controlled by custom software developed using Matlab (The Mathworks, Natick, MA).

### 2.3 Training

Naïve animals took about a week to learn the two-alternative forced-choice task. During the initial training, the animal had to insert its nose into the center spout poke-hole for 200 ms after which a broadband noise burst was played from one of the speakers. In order to receive a reward, the animal approached the source of the sound and placed its nose into the underlying poke-hole containing the response spout. Once the animal learnt the mechanics of this task and performed ∼100 trials on each session, we increased the delay at the center spout (in the final experiments to 1–7 s, in order to ensure that the animal waited for the sound to be presented) and started training the animals by playing broadband noise from both speakers simultaneously but with a difference in level of 30 dB between them. The animal then had to judge the perceived location of the sound by licking the response spout on the side of the loudest sound. To avoid a bias towards one speaker location, an incorrect response was followed by a number of correction trials (same stimulus and location) until the animal responded correctly. Once the animals attained a stable performance of >95% correct for several days, they were judged trained and ready for implantation of the cranial support.

After implantation, the animals were tested again for their ability to discriminate between the same free-field noise bursts, and were then presented with these stimuli over headphones using Panasonic headphone drivers (RP-HV280, Panasonic, Bracknell, UK) across which the interaural level difference (ILD) was varied, as outlined in Section 3. The average binaural level of the closed-field stimuli was varied from 70 to 80 dB SPL.

### 2.4 Surgical procedures

After overnight food deprivation, the animals were anesthetized by an intramuscular administration of a mixture of 0.022 mg/kg medetomidine hydrochloride (Domitor; Pfizer Ltd, Kent, UK) and 5 mg/kg ketamine (Ketaset; Fort Dodge Animal Health, Southampton, UK). The left radial vein was cannulated for drug and fluid (saline 5 ml/h) administration, the trachea was intubated for mechanical ventilation and the anesthesia was switched to isoflurane 0.5–1.5% (IsoFlo; Abbott Laboratories Ltd, Kent, UK). Throughout the procedure the temperature, ECG and end tidal CO_2_ were monitored and maintained. Once the animal was stabilized, the following drugs were administered intramuscularly: atropine (0.006 mg/kg. Atrocare; Animalcare Ltd, York, UK) to reduce secretions; buprenorphine (0.03 mg/kg. Vetergesic; Alstoe Animal Health, Melton Mowbray, UK) and meloxicam (0.2 mg/kg. Metacam; Boehringer Ingelheim, Terrassa, Spain) to provide perisurgical analgesia. We administered atipamezole (Antisedan; Pfizer Animal Health, Kent, UK) subcutaneously to reverse the effect of medetomidine once the animal was switched to isoflurane anesthesia. Finally, local anesthetic (Elma; Astra Zeneca Luton, UK) was applied to the stereotaxic pressure points and carbomer (Viscotears; Lewis Pharmaceuticals Ltd, Doncaster, UK) to the eyes.

Once the head was fixed in the stereotaxic frame, a midline incision, typically 30–40 mm in length, exposed the dorsal part of the skull and the temporal muscles were partially displaced laterally. The exposed skull surface was cleaned mechanically of any soft tissue and etched using a 1% citric acid solution. In two animals, up to four steel self-tapping screws were attached to the skull. In the remaining five animals, no screws were used.

A thin layer (∼1 mm thick) of dental adhesive (Super-bond C&B; Sun Medical Co, Shiga Japan) was applied to the area of the skull where the cranial pedestal (see Section 3) was to be built using bone cement (CMW1 Bone Cement; DePuy CMW, Lancashire, UK). Typically the area covered by the implant was about 3 cm^2^, expanding 20 mm rostrally from the back of the skull with a lateral width of 15 mm centered at the midline. Once the layer of dental adhesive, prepared following the manufacturer's instructions, had formed a skin, the first layer of bone cement (1–2 mm thick) was poured over it. Additional bone cement was then applied to raise the implant while reducing its area up to final dimensions of ∼1.5 mm × 10 mm at the height of the original level of the skin (∼4 mm at the midline). This approach maximized the bonding area of the implant, whilst minimizing the region to be externalized above the scalp. Consequently, we were able to reposition the temporal muscles without having to trim them. Due to the highly exothermic curing of the bone cement, the implant was built in small layers, allowing complete hardening and cooling of the previous layer before applying the next one to avoid heat damaging the underlying bone.

Once the part of the cranial implant that was to lie under the skin was finished, the temporal muscles were repositioned and stabilized by suturing them at the lateral aponeuroses at the back of the skull and sutured together in front of the implant. Following this procedure, the muscles reattached to the skull and to the most lateral and deepest aspects of the implant. The skin was sutured at the midline in front of and behind the implant after being trimmed using scissors around the implant. Once the skin was sutured the external part of the implant was constructed to allow attachment of the headphones (see Section 3 for details). When the implant was finished and fully cured, the animal was removed from the stereotaxic frame, the incision to cannulate the radial vein (<7 mm) stitched, and, once recovered from anesthesia, returned to its cage.

A post operative analgesic protocol was followed for 5 days, comprising 3 days of treatment with buprenorphine and meloxicam followed by two more days of treatment with meloxicam. Because the surgery was carried out in sterile conditions, no prophylactic or perioperative antibiotic was deemed to be required, and no signs of infection were observed in any animal after surgery. Before starting the training with headphones, we allowed complete healing of the surgical incision and the skin surrounding the implant.

### 2.5 Acoustical measurements

Acoustical measurements were carried out in an anechoic chamber using a ferret cadaver that underwent the same implantation procedure as that adopted for the animals that were used for behavioral testing. The ferret cadaver was situated at the center of the anechoic chamber and a pair of polythene tubes (o.d. 1.52 mm, i.d. 0.86 mm) were inserted at the entrance of the ear canal and connected to a pair of Sennheiser microphone capsules (KE-4-211-2; Wedemark-Wennebostel, Germany) via customized connectors. The transfer function of each earphone was measured, flattened up to 35 kHz, and calibrated to 85 dB SPL. Test stimuli were 1000-ms bursts of broadband noise presented at 85 dB SPL and were presented unilaterally over headphones attached to the implant. All stimuli were generated and recorded using an RP2 running at 100 kHz, controlled via customized Matlab code. Interaural attenuation was calculated by comparing the acoustical responses measured at each ear via implanted probe-tube microphones at the entrance of the ear cannal. The same probe-tube microphones were additionally used to record the acoustical response to a 1000-ms burst of broadband noise flattened up to 20 kHz, and presented at 85 dB SPL from a location 1 m directly in front of the animal. We then attempted to replicate this response using appropriately filtered closed-field stimuli. The Fourier transform of the free-field response (*F*_ffr_) is determined by the transfer functions of the head (*F*_hrtf_), the ear canal (*F*_ec_), and the probe-tube microphone (*F*_m_),

(1) $$F_{\text{ffr}}=F_{\text{hrtf}}\times F_{\text{ec}}\times F_{\text{m}}\text{,}$$

while the Fourier transform of the closed-field response (*F*_cfr_) is determined by the transfer functions of the earphone (*F*_e_), the ear canal (*F*_ec_), and the probe-tube microphone (*F*_m_),

(2) $$F_{\text{cfr}}=F_{\text{e}}\times F_{\text{ec}}\times F_{\text{m}}\text{,}$$

Consequently, the filter required for simulating the free-field response using closed-field stimulation (*F*_s_) can be obtained by dividing the Fourier transform of the free-field response by that of the closed-field response,

(3) $$F_{\text{s}}=\frac{F_{\text{ffr}}}{F_{\text{cfr}}}$$

and generating a minimum-phase filter with the resulting transfer function.

## 3 Results

The purpose of this study was to develop a detachable headphone system that allows reliable closed-field acoustic stimulus presentation in freely moving animals. The fidelity of this system was assessed by measuring the behavioral responses to stimuli presented with a range of ILDs in ferrets that had previously been trained on a lateralization task with free-field stimulation. All animals tested readily transferred the learnt behavior between the free-field and closed-field stimulation conditions.

### 3.1 Design of the earphone holder

**Fig. 1:**
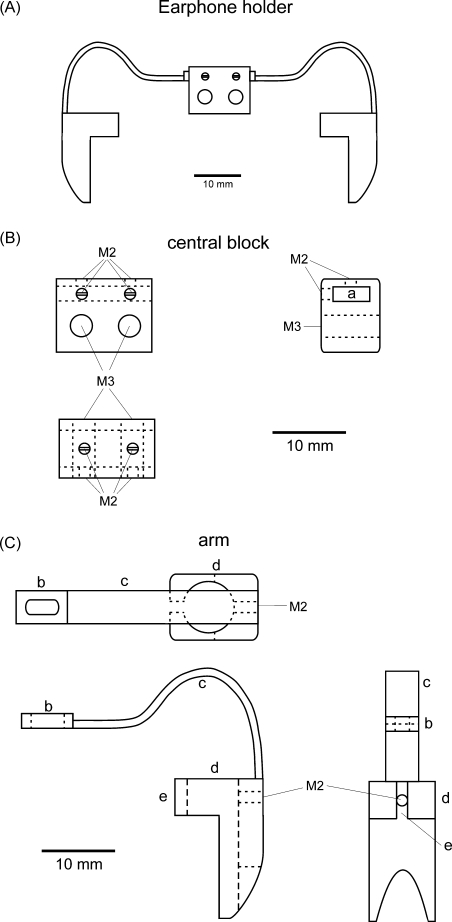
Drawings of the headphone system. (A) The assembled headphone, which consists of a central block (detailed in B) and two arms to hold the earphones (detailed in C). The central block has two threads of 3 mm diameter (M3) to allow its fixation to the chronic pedestal implanted on the animal's skull (see Fig. 3). Both earphone holder arms are inserted in the slot that runs through the block (a) and adjusted and secured using 4 bolts of 2 mm in diameter (M2). A slot at the end of each arm (b) allows them to be secured by the bolts (M2) at the top of the central block. On the other end of the arm is the earphone holder (d), which has an opening (e) through which the cable for the earphone is passed and a 2 mm bolt (M2 in C) to secure it. The thin middle part of the arms (c), while strong enough to prevent accidental bending by the animals, is sufficiently flexible to allow the position of the earphones to be adjusted so that they are aligned with the ears, thereby taking into account individual variations in head size and/or head implants.

The earphone holder (Fig. 1) was made of titanium so that its weight could be kept to just 11.5 g. It consisted of a central block (Fig. 1B) with two M3 threads to allow fixation to the skull-mounted pedestal by means of two M3 bolts, and two arms (Fig. 1C) in which earphones were slotted in and secured by M2 bolts.

**Fig. 2:**
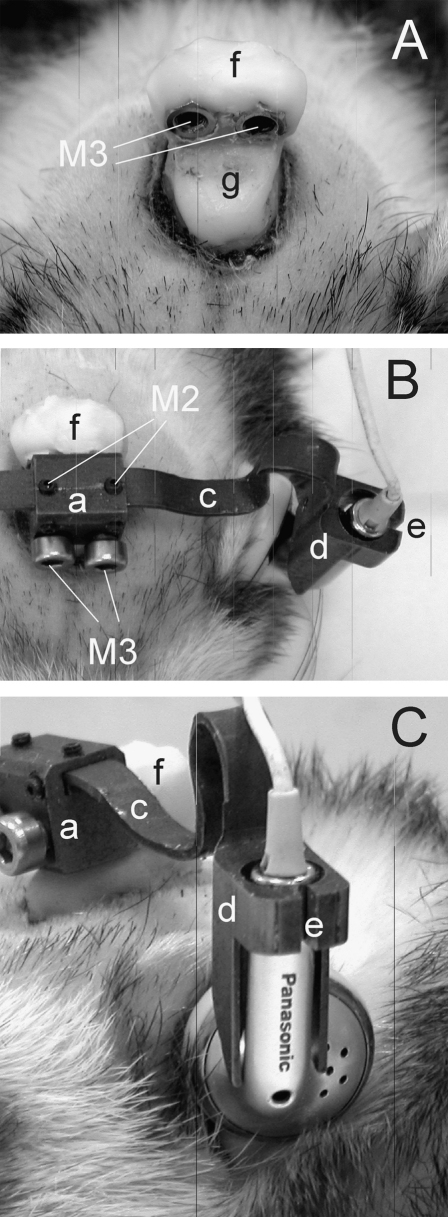
Close up photographs of a chronic implant (A) and of an animal wearing the headphones (B, C). (B) Dorsal view. (C) Lateral view. Headphones are attached to the bone cement pedestal (f, g), which has two hexagonal nuts embedded so that the central block of the headphones (a) could be secured to it by two 3 mm bolts (M3). The arms of the headphones (c) can be adjusted via the 2 mm bolts (M2) to bring the earphones close to the entrance of the ear canal. Other labels: d, earphone holder; e, opening in the holder, through which the cable is passed so that the earphone can be inserted.

Because detachability was one of our requirements, we opted to have two anchor points instead of one to ensure consistent positioning of the earphones across different sessions, avoiding the possibility of rotations that could arise from a single fixation point (Fig. 1A and B). A slot was included at the end of each arm, through which M2 bolts were passed to attach it to the central block (Fig. 1C). This enabled the arms, which consisted of thin curved strips of titanium, to be moved in or out to allow for individual differences in head size or to correct for small deviations from the midline that could occur during the building of the implant. The bend in the middle part of each arm could be adjusted easily using pliers to ensure that the earphones were positioned correctly at the entrance of the ear canal (Fig. 2). Importantly, however, the arms were sufficiently rigid to prevent accidental bending caused by the animal during normal use.

### 3.2 Chronic pedestal

In the first two animals, in addition to the dental adhesive we used up to four steel self-tapping screws to ensure good fixation of the implant to the skull. Those animals wore their chronic implants for one year and during that time no infection developed around the implant. Once the skin healed around the implant (1–2 weeks postsurgery) no cleaning or special care was needed apart from the occasional hair trimming around the implant to keep it visible. The ready acceptance by these two animals to wear the headphones, together with the superior strength of those initial implants compared to the ones used in acute recording experiments, where fixation screws only are used, encouraged us to abandon the use of screws in the remaining animals. The five animals in which no screws were used were implanted in July 2009 and, eight months later, are still yielding useful data without observing any weakening of the bond between the pedestal and the bone, indicating that the dental adhesive is sufficient by itself for providing long-lasting attachment of the implant.

We built the pedestal out of bone cement in which two hexagonal nuts (3 mm thread (M3) and 6 mm long) were embedded to allow fixation to the central block of the earphone holder (Fig. 2). To ensure their correct alignment, we bolted the nuts to the central block and held them in position with a manipulator while surrounding them with bone cement until this was completely cured. In contrast to implanting a metal post or other device embedded in the pedestal, the use of two nuts not only reduced the weight of the implant to <5 g, but also saved us from having to design and produce small custom parts that are generally not reusable and frequently require fitting to accommodate individual differences in skull morphology (Dobbins et al., 2007). This approach also reduced the duration of the surgery by simplifying the pedestal construction, particularly after we found that skull fixation screws were not required.

To increase the area of adhesion, the temporal muscles were partially detached from the midline and retracted temporally so that a thin layer of bone cement could be applied to spread the load. However, the pedestal was built up in height and externalized only near the midline (Fig. 2). This allowed the intact temporal muscles to be repositioned, reducing the duration of postoperative recovery. In order to lower the risk of infection, the visible part of the pedestal was built once the scalp was trimmed and reattached with sutures in front of and behind the implant. Because the arms of the headphones were removable, only the central block was required for aligning the two hexagonal nuts while embedding them in the bone cement.

### 3.3 Acoustical measurements

**Fig. 3:**
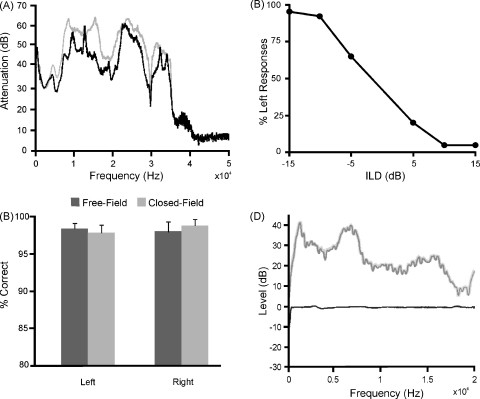
(A) Sound attenuation in the ear contralateral to the stimulus plotted as a function of frequency with (in gray) and without (in black) an earplug in the contralateral ear. These measurements were taken with the headphones attached in the same manner used for behavioral testing. (B) Percentage of responses made to the left side in one animal following presentation of 200-ms noise bursts over the headphones at the ILDs indicated. (C) Comparison of percentage correct scores elicited by free-field and closed-field conditions for five animals, with average binaural level roved between 70 and 80 dB SPL. In the closed-field condition, the ILD was ±5 dB. (D) Spectral envelope of a free-field stimulus positioned 1 m in front of the animal is shown in light gray, as recorded using a probe-tube microphone inserted into the ear canal of a ferret. Spectral envelope of an appropriately filtered closed-field stimulus, recorded using the same probe-tube microphone, is shown in dark gray, with the difference between the free-field and closed-field spectra shown in black. For visualisation purposes, the mean dB SPL value across frequency was calculated for the free-field spectrum and deducted from both the closed- and free-field spectra.

Several stimulus presentations were made to measure the interaural attenuation while wearing the headphones. Between presentations the headphones were removed and reattached to the chronic pedestal to account for the possibility of changes due to the repositioning of the headphones. Interaural attenuation was typically around 40 dB up to the maximum frequency tested (35 kHz), with a minimum value of approximately 20 dB (Fig. 3A). Although the introduction of an earplug increased interaural attenuation at specific frequencies, these increases were relatively modest and were almost non-existent at frequencies below <1.5 kHz. This suggests that attempts to further acoustically seal the ears would produce little additional benefit, particularly at low frequencies where crosstalk is most likely mediated by mechanisms related to bone conduction. This therefore enabled us to measure the ILD sensitivity of the ferrets (over a ±15 dB range) without any major influence of crosstalk.

Although attachment of the headphones to the cranial implant via two bolts ensured that the earphones were reliably positioned over the ears, it was still important to assess the consistency of the probe-tube microphone measurements with repeated attachment of the headphones. Under conditions approximating normal use, these measurements showed that the overall sound level recorded in each ear typically remained consistent to within 1 dB (SD = 1.1 dB).

To illustrate the level of stimulus control made possible by this system, and to demonstrate its suitability for virtual acoustic space techniques, we used probe-tube microphones to record the acoustic response to a free-field stimulus and attempted to replicate this response using closed-field stimuli that had been appropriately filtered (see Section 2). Fig. 3D shows that these attempts were highly successful, with errors of generally less than 1 dB across all frequencies tested. Although we only attempted to match the acoustic response in one ear, this approach is easily extended to both ears, confirming the suitability of this system for VAS studies. More generally, these data show that digital filtering techniques can be used to control the spectrum of the stimulus available at the eardrum, facilitating the presentation of arbitrary spectra associated with complex sounds.

### 3.4 Behavior with headphones

After a sufficient period of recovery following implantation of the cranial pedestal, the animals were retested in the free-field task to ensure that they were performing at the same level as before the surgery. At the start of each closed-field stimulation session, the earphone holder was attached, having previously adjusted the arms so that the earphones were positioned appropriately. The earphones made light contact with the pinnae, their soft pads ensuring that any discomfort to the animal was minimized. The earphone cables exited through the center of the ceiling of the behavioral chamber and were attached to it to ensure that no restriction was imposed on the movement of the animal. After the first few days, the behavior of the ferrets appeared to be completely unaffected by the presence of the headphones and the cables.

The animals were initially trained using randomly interleaved ILDs of ±15 dB. Their performance on this fixed-ILD lateralization task steadily improved over the course of a few weeks, most likely as they became acquainted with wearing the headphones, and reached a consistent level of performance of >98% correct. They were then tested with a range of smaller ILDs, and an example of the data and the fitted psychometric function obtained at an early stage of training from one animal is shown in Fig. 3B. The mean percentage correct scores achieved by all the animals are plotted in Fig. 3C, along with their performance on the free-field version of the task in which the stimulus was presented from either the left or the right loudspeaker. For both free-field and closed-field versions of the lateralization task, the mean scores were again ∼98% correct and the standard deviations were very low, indicating that all the animals were able to judge the side from which the sound originated on the basis of the headphone signals to a high degree of accuracy. Moreover, no differences were found in either the free-field or the ILD data in the scores measured at different average binaural levels (paired *t*-test, *P* = 0.748). This shows that the fidelity of our sound delivery system is sufficient to support performance of an ILD task that is as good as that observed under free-field conditions, and suggests that, in the closed-field condition, the ferrets were lateralizing the stimulus on the basis of the ILDs rather than monaural differences in sound level.

## 4 Discussion

The detachable headphones that we have developed allow sounds to be presented dichotically to freely moving animals, thereby facilitating the behavioral measurement of sensitivity to different sound localization cues. We have shown that ferrets adapt quickly to wearing the headphones and perform to the level expected when ILDs are provided over the headphones, indicating that the animals are readily able to transfer the spatial behavior learnt from free-field to closed-field stimulation.

### 4.1 Practical considerations

One of the main issues when designing the headphones was the welfare cost for the animal. This was reduced in a number of ways by (1) minimization of the size and weight of the headphones and cranial support, (2) removing the headphones to facilitate normal behavior when not in use, (3) the ease and consistency of headphone positioning and attachment, and (4) limiting the invasiveness of the surgery required for implantation.

The total weight borne by the animals was ∼23 g (earphone holder 11.5 g, earphones 6 g, and chronic implant <5 g), but <5 g when the headphones were removed. Once adjustments are made for head size, this design should be suitable for a number of other species, such as non-human primates, cats and rats. All the ferrets used in this study adapted quickly to wearing the headphones and, beyond the first 1–2 min following attachment did not try to remove them. No obvious postural change was observed as a result of wearing the headphones, which has so far been limited to about one hour daily divided into two or three sessions. Importantly, it was not necessary to use a commutator to enclose the ceiling-mounted cables as we have observed no instances of an animal becoming caught or entangled in them. This obviously has considerable implications for the cost of this setup. Although the method of tethering the animal might be more of an issue in a larger testing arena, boxes of the approximate size used in this study are perfectly adequate for measuring the discrimination abilities of ferrets in a range of auditory tasks (Walker et al., 2009; Bizley et al., 2009; Keating et al., 2010).

The modest size of the cranial pedestal meant that chronically implanted animals could be housed in the same conditions and shared cages with other ferrets, without fear of damage to the implant. Antibiotic treatment is normal practice in animals with chronic implants (e.g. Mickey and Middlebrooks, 2003; Adams et al., 2007; Dobbins et al., 2007). However, because of the stability of the interface between skin and implant, no special cleaning was required beyond the occasional trimming of the surrounding hair to keep it visible and facilitate the headphone attachment. Moreover, no antibiotic treatment was required as we have seen no instances of infection.

Although our headphone system still requires surgical intervention for building the chronic implant, the surgery time has been shortened by moving away from traditional methods of skull fixation, and using a dental adhesive instead. Because of the thinness of the skull, adhesive resins have been adopted for attaching cranial implants in small animals (Okumura et al., 2007), whereas anchoring bone screws tend to be used in larger species (e.g. Mickey and Middlebrooks, 2003; Adams et al., 2007; Dobbins et al., 2007). The latter can be associated with complications such as infection or damage of the underlying neural tissue. Consequently, our finding that dental adhesive alone can provide enough structural stability to hold a lightweight implant in place in adult ferrets for at least several months has important implications for other studies of this sort.

Because we have not directly compared the strength of the cranial implant in the presence and absence of self-tapping screws, it is unclear how much additional stability would be provided by the use of screws. For our purposes, however, self-tapping screws were unnecessary, but it is difficult to extrapolate to other preparations because of variability in (1) the composition and size of the implants, (2) the forces the implants are exposed to, and (3) the characteristics of the bone supporting the implant, all of which will ultimately depend on the species and degree of restraint required. Although there are data on the shear bond strength of different dental adhesives, including the one used in this study, to dentine (Altintas et al., 2008; Yang et al., 2006), we are not aware of any comparable information on the strength provided by more traditional methods of fixation. It should be noted, however, that we have observed no incidences of implant detachment over an 8-month period, whereas the failure rate has been reported to be as high as 20% in other studies where bone screws were used (Ward et al., 2009). We suggest that the use of adhesive, a common practice in dentistry, should be considered for any cranial implant, particularly in species with thin skulls, as the preferred method of fixation, either by itself or in combination with bone screws.

### 4.2 Behavioral measures of binaural sensitivity

It is possible to measure the spatial sensitivity of auditory neurons by situating earphones close to, but not actually touching, the opening of the animal's ear canals (Behrend et al., 2004), thereby avoiding any potential discomfort caused by direct contact with the pinnae. However, we considered it more appropriate, particularly in a freely moving animal, to position the earphones so that they were directly apposed to the ears but fully supported by the cranial implant. This does not restrict the animal's natural orienting response, as, in contrast to species like cats, ferrets do not make directional pinna movements. A similar approach was used by Otazu et al. (2009), who placed earphones over the pinnae in freely moving rats via a pair of head-mounted plastic rings.

The close proximity of the sound source to the head maximizes the acoustic shadowing effect (Akeroyd, 2006), with the result that the crosstalk attenuation between the two earphones was ∼40 dB, which is larger than the maximum ILD generated by the ferret's head and external ears (Carlile and King, 1994). There was therefore no need to seal the earphones into the ear canals, which would have added to both the setup time at the start of each behavior session and the discomfort for the animals. The two point attachment of the holder to the chronic implant ensured consistency in the positioning of the earphones, which was confirmed by the reproducibility of the behavioral and acoustical measurements.

In humans, dichotic sounds are increasingly lateralized as the ILD is increased up to a value of ∼10 dB (Akeroyd, 2006). Similarly in the ferret, with an ILD of ±5 dB, we registered performances of near 100% correct. We showed that it is possible to obtain a complete psychometric function within a single testing session, paving the way for behavioral and physiological measurements of the animals’ sensitivity to different localization cues, and of how that sensitivity changes with experience and learning. Another advantage of having a closed-field stimulation system is the possibility of combining behavioral studies with virtual acoustic space stimuli that simulate real sounds over headphones, an approach that, as far as we are aware, has only been possible in humans (Wightman and Kistler, 1992; Jin et al., 2004) and barn owls (Hausmann et al., 2009).
